# Correlation of *RUNX2* Variants With Craniofacial–Dental Phenotypes in Cleidocranial Dysplasia

**DOI:** 10.1002/cre2.70351

**Published:** 2026-04-17

**Authors:** Pintu‐on Chantarawaratit, Sermporn Thaweesapphithak, Pisha Pittayapat, Thantrira Porntaveetus

**Affiliations:** ^1^ Department of Orthodontics, Faculty of Dentistry Chulalongkorn University Bangkok Thailand; ^2^ Center of Excellence in Precision Medicine and Digital Health, Geriatric Dentistry and Special Patients Care Program, Faculty of Dentistry Chulalongkorn University Bangkok Thailand; ^3^ Department of Oral Biology, International College of Dentistry Walailak University Bangkok Thailand; ^4^ Department of Radiology, Faculty of Dentistry Chulalongkorn University Bangkok Thailand; ^5^ Clinic of General, Special Care and Geriatric Dentistry, Center for Dental Medicine University of Zurich Zurich Switzerland

**Keywords:** cleidocranial dysplasia, craniofacial morphology, embedded teeth, genotype–phenotype correlation, *RUNX2*, skeletal Class III malocclusion, supernumerary teeth

## Abstract

**Objectives:**

Cleidocranial dysplasia (CCD) is a rare *RUNX2*‐related skeletal disorder characterized by craniofacial anomalies and skeletal Class III malocclusion. However, the relationship between *RUNX2* variant type and phenotype severity remains unclear. This study aimed to evaluate the association between *RUNX2* variant types and the severity of skeletal Class III malocclusion and dental anomalies in CCD.

**Material and Methods:**

This cross‐sectional study included 11 unrelated Thai CCD probands and 14 relatives who underwent exome or genome sequencing. *RUNX2* variants were classified as non‐truncating or truncating/structural according to ACMG‐AMP criteria. Nine genetically confirmed individuals met the inclusion criteria for radiographic analysis. Craniofacial parameters and dental anomalies were assessed using lateral cephalograms and cone‐beam computed tomography. Group comparisons were performed using nonparametric tests and correlation analysis.

**Results:**

Truncating/structural variants were associated with shorter cranial base length, smaller SNA, and reduced nasolabial angles compared with non‐truncating variants. The variant type correlated with these parameters. Skeletal Class III malocclusion was present in both groups, predominantly due to mandibular prognathism. No significant association was found between variant type and the burden or distribution of dental anomalies.

**Conclusions:**

*RUNX2* variant type influences cranial base and maxillary morphology, contributing to skeletal Class III severity in CCD, whereas dental anomalies appear independent of variant type.

## 1 Introduction

Cleidocranial dysplasia (CCD) is an exceptionally rare skeletal disorder, with an estimated global prevalence of approximately 1 in 1,000,000 individuals (Machol et al. 2006). It follows an autosomal dominant inheritance pattern and results primarily from variants in the *RUNX2* gene, which encodes a transcription factor essential for osteoblast differentiation and normal skeletal development (Otto et al. 2002; Ryoo et al. 2010; Thaweesapphithak et al. 2024). The *RUNX2* protein contains several functional domains, including the Runt domain, responsible for DNA binding and heterodimerization, and the PST (proline/serine/threonine‐rich) domain, which plays a critical role in transcriptional activation. Variants in these domains can impair protein function to varying degrees, leading to defective ossification and a spectrum of craniofacial and dental abnormalities (Machol et al. 2006; Otto et al. 1997; Thaweesapphithak et al. 2023). While the classical skeletal features of CCD include delayed closure of cranial sutures and hypoplastic or absent clavicles, the dental manifestations are equally complex and clinically significant (Jaruga et al. 2016; Jensen 1994; Machol et al. 2006; Thaweesapphithak et al. 2022).

From an orthodontic standpoint, CCD is particularly notable for its severe dental anomalies. Patients frequently present with multiple impacted permanent teeth, numerous supernumerary teeth, embedded teeth, and prolonged retention of the primary dentition (Camilleri and McDonald 2006; McNamara et al. 1999). The presence of excessive supernumerary teeth often results in severe crowding and dense packing of teeth within the alveolar bone, causing mechanical obstructions that hinder normal eruption of the permanent teeth into the oral cavity (Ryoo et al. 2010). These factors contribute to a poor response to conventional eruption guidance and often necessitate interdisciplinary treatment approaches, including surgical‐orthodontic interventions. Additionally, most CCD patients demonstrate a skeletal Class III malocclusion, frequently exhibiting a severity that necessitates orthognathic surgical intervention (Faria‐Teixeira et al. 2024; Jaruga et al. 2016; Jensen 1994). These craniofacial characteristics further complicate both diagnosis and treatment planning, particularly in growing patients, where the timing of intervention is critical.

Although the genetic basis of CCD has been well established, the relationship between specific *RUNX2* variants and the severity of craniofacial and dental phenotypes remains poorly understood. Previous research in CCD has identified a wide range of phenotypes, but a systematic investigation into genotype–phenotype correlations is lacking (Lee et al. 2015; Thaweesapphithak et al. 2024). While some studies have suggested links between specific *RUNX2* variants and certain features of CCD, a comprehensive understanding of the relationship between genetic variants and the diverse clinical presentation remains incomplete (Thaweesapphithak et al. 2022; Thaweesapphithak et al. 2024). Most prior reports have been limited to descriptive case series or focused on general skeletal features without quantitative assessment of occlusal severity or dental anomalies (Chen et al. 2023; Farronato et al. 2009; Jaruga et al. 2016; Jensen 1994; Park et al. 2013; Rocha et al. 2014; Thaweesapphithak et al. 2022). To date, no study has specifically examined whether the type or location of *RUNX2* variants correlates with the severity of skeletal Class III malocclusion or the burden of embedded teeth, both of which are highly relevant to clinical management (Jensen and Kreiborg 1990; Kreiborg and Jensen 2018).

Identifying such genotype–phenotype correlations could enhance early diagnosis, risk stratification, individualized treatment planning, and genetic counseling for affected families (Machol et al. 2006). No study has systematically investigated genotype–phenotype correlations between *RUNX2* variant types and quantitative cephalometric parameters in patients with CCD. This study is the first to integrate molecular genetic data with cephalometric analysis to elucidate craniofacial phenotype severity in this rare skeletal disorder, particularly in the Southeast Asian population. Therefore, the objective of this study is to investigate the relationship between *RUNX2* gene variants and craniofacial as well as dental phenotypes in patients with CCD. Specifically, this study aims to determine whether different types or locations of *RUNX2* variants are associated with (1) the severity of skeletal Class III malocclusion and (2) the number and distribution of embedded and supernumerary teeth. The null hypothesis is that there is no significant association between the type or location of *RUNX2* variants and the severity of skeletal Class III malocclusion, the number and distribution of embedded and supernumerary teeth, or cephalometric skeletal measurements in individuals with CCD.

## 2 Materials and Methods

This study was designed as an observational cross‐sectional study to evaluate the association between *RUNX2* variant types and craniofacial–dental phenotypes in patients with cleidocranial dysplasia.

### 2.1 Subject Enrollment

**FIGURE 1:**
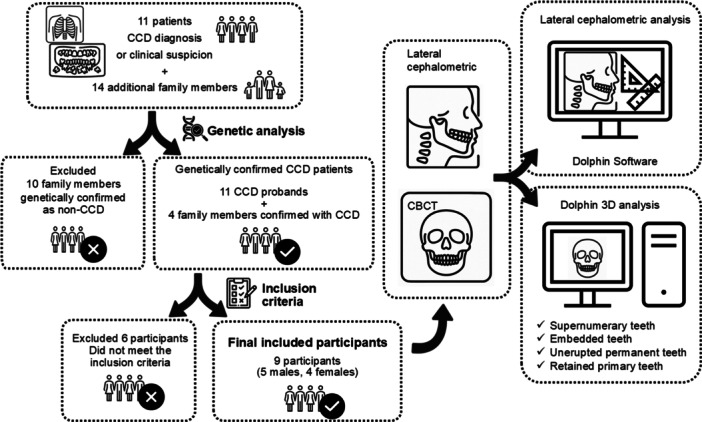
Schematic illustration of the subject enrollment process and study protocol.

The subject enrollment and study protocol are shown in Figure 1. Eleven unrelated Thai individuals who were either clinically diagnosed with or under clinical suspicion of CCD, along with 14 additional family members, were consecutively recruited from patients attending the Faculty of Dentistry, Chulalongkorn University, between July 2021 and November 2025. Eligible patients were identified during routine clinical visits based on predefined diagnostic criteria. Given the rarity of cleidocranial dysplasia, a formal a priori sample size calculation was not feasible. Therefore, all eligible patients who presented during the study period were consecutively recruited.

The study was approved by the Human Research Ethics Committee (HREC‐DCU 2021‐030/2025‐141) and complied with the Declaration of Helsinki. The clinical diagnostic criteria for CCD include: (a) hypoplasia or aplasia of the clavicles; (b) a prominent forehead, enlarged fontanels, or delayed closure of the anterior fontanel; and/or (c) retention of primary teeth, failure of eruption of permanent teeth, and the presence of supernumerary teeth (Farrow et al. 2018; Machol et al. 2006; Mundlos 1999; Thaweesapphithak et al. 2022). A thorough investigation and blood sample collection were conducted after obtaining written informed consent from all participants or their legal guardians (Thaweesapphithak et al. 2022).

### 2.2 Genetic Analyses

Genomic DNA was extracted from 3 mL of peripheral blood leukocytes collected from the probands, their available family members and healthy individuals (as controls). Variant analysis was conducted using either exome sequencing or genome sequencing.

For exome sequencing, genomic DNA was captured using the SureSelect Human All Exon version 4 kit (Agilent Technologies, Santa Clara, CA, USA) and sequenced on the HiSeq. 2000 platform (Macrogen, Seoul, South Korea). Sequence reads were aligned to the human reference genome (UCSC hg19) using the Burrows‐Wheeler Aligner (BWA; http://bio-bwa.sourceforge.net/). Subsequent data processing was performed using SAMtools (http://samtools.sourceforge.net/), and annotations were derived from dbSNP and the 1000 Genomes Project databases.

For genome sequencing, genomic DNA was sequenced on the BGI DNBSEQ‐T7 platform, achieving an average coverage depth of 30x. Reads were aligned to the human reference genome (GRCh38) using the Burrows‐Wheeler Aligner with the Maximal Exact Match (BWA‐MEM). Single‐nucleotide variants (SNVs) and insertions/deletions (indels) were called using the Genome Analysis Toolkit (GATK). Copy number variants (CNVs) and structural variants (SVs) were identified from the aligned reads (BAM files) using CNVkit and MANTA software, respectively.

Variants with a minor allele frequency (MAF) greater than 1% in the 1000 Genomes Project or Genome Aggregation Database (gnomAD), or present in more than 10 alleles in our in‐house database of 3206 Thai exomes, were excluded.

The potential pathogenicity of the variants was evaluated using in silico prediction tools, including PolyPhen‐2, SIFT, MutationTaster, CADD, and REVEL, based on their predicted impact on protein structure and gene function, and/or their association with the patients' phenotypes. Variant classification adhered to the guidelines of the American College of Medical Genetics and Genomics and the Association for Molecular Pathology (ACMG‐AMP). Variants were considered novel if not previously reported in ClinVar (https://www.ncbi.nlm.nih.gov/clinvar) or gnomAD (https://gnomad.broadinstitute.org/).

Genetic analysis confirmed 11 patients as probands, with an additional four family members definitively diagnosed with CCD (six males and nine females; mean age: 30.9 ± 20.4 years; range: 5–77 years). Then, participants were eligible for inclusion in the radiographic analysis if they had a genetically confirmed *RUNX2* variant, were aged ≥ 13 years for females or ≥ 15 years for males, to ensure adequate dental and craniofacial maturity for reliable assessment (Burstone 1963; Manlove et al. 2020), and had no history of orthodontic treatment, orthognathic surgery, tooth extraction, or surgical removal of embedded teeth that could affect craniofacial or dental structures. Participants were excluded if they were below the specified age thresholds, had undergone any dental or surgical interventions that could alter craniofacial morphology and had incomplete or poor‐quality radiographic records, or presented with other craniofacial syndromes, skeletal disorders, or pathological lesions that could confound phenotypic evaluation. As a result, nine participants (five males and four females; mean age: 27.4 ± 9.0 years; age range: 15–45 years) were included for further radiographic analysis. The demographic and genetic characteristics of the participants are shown in Table 1.

**TABLE 1:** Demographic and genetic characteristics of study participants.

Patients	Family	Sex	Age	Gene	Mutation	Exon	Domain	Type of variant	Groups	Novel
Case 1	Proband	M	36	*RUNX2*	Microdeletion	3′UTR	—	Microdeletion	2	Novel
Case 2	Proband	F	20	*RUNX2*	c.673 C > T, p.Arg225Trp	4	RUNT	Missense	1	Known
Case 3	Proband	M	21	*RUNX2*	c.1081 C > T,p.Gln361Ter	7	PST	Nonsense	2	Known
Case 4	Proband	F	28	*RUNX2*	c.739delA, p.Ser247ValfsTer3	4	PST	Frameshift InDel	2	Known
Case 5	Proband	F	25	*RUNX2*	Microdeletion	1‐4	QA and RUNT	Microdeletion	2	Novel
Case 6	Proband	F	15	*RUNX2*	c.614 C > T, p.Thr205Ile	3	RUNT	Missense	1	Novel
Case 7	Proband	M	26	*RUNX2*	c.1550_1553del, p.Val517Glyfs*61	9	VWRPY	Frameshift InDel	2	Novel
Case 8	Proband	M	31	*RUNX2*	c.90dup, p.Ser31Leufs*130	1	—	Frameshift InDel	2	Known
Case 9	Family	M	45	*RUNX2*	c.614 C > T, p.Thr205Ile	3	RUNT	Missense	1	Novel

### 2.3 Radiographic Analysis

#### 2.3.1 Lateral Cephalometric Analysis

The cephalometric landmarks and measurements utilized in this study are detailed in Supporting Information S1: Tables 1 and 2 (Duangsuwan et al. 2023; Kapila and Nervina 2015; Sorathesn 1988). Lateral cephalograms were obtained from CS9000C extraoral imaging system (Carestream Rochester, NY, USA) at 74 kVp and 10 mA, with an exposure time of 0.50 s and a source‐to‐image distance of 5 feet. Subjects were positioned upright with the neck straight, teeth in maximum intercuspation, and lips at rest without muscle strain. All files were imported into Dolphin 3D software version 11.9 Premium (Dolphin Imaging & Management Solutions, Chatsworth, CA, USA) for processing. Cephalometric landmarks were manually identified, and linear and angular measurements were automatically calculated based on the predefined cephalometric analysis selected in the program using the 2D Cephalometric Analysis Module in Dolphin software. A total of 14 cephalometric parameters, including four linear and 10 angular measurements, were evaluated according to conventional cephalometric analysis standards. To ensure measurement accuracy, the software was calibrated using a known reference distance prior to analysis. All landmark identifications and measurements were performed by a single examiner experienced in craniofacial radiographic analysis.

#### 2.3.2 Data Collection of Dental Anomalies: Embedded Teeth, Supernumerary Teeth, Unerupted Permanent Teeth, and Retained Primary Teeth

Cone‐beam computed tomography (CBCT) images were obtained using a 3D Accuitomo 170 unit (J. Morita Corp., Kyoto, Japan) with a voxel size of 0.25 mm, operating in standard mode at 90 kVp and 5 mA, with an exposure time of 30.8 s. All scans were performed with patients in a natural head position and included the midface, maxilla, and mandible.

**FIGURE 2:**
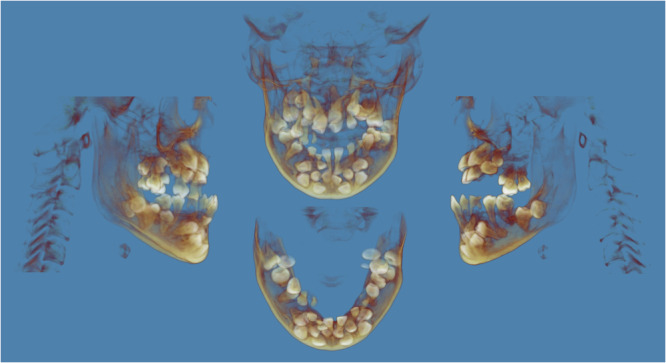
Three‐dimensional reconstructions generated using Dolphin 3D software (Dolphin Imaging & Management Solutions, Chatsworth, CA, USA) illustrating supernumerary teeth, embedded teeth, unerupted permanent teeth, and retained primary teeth identified during the data collection of dental anomalies. The images can be presented from multiple perspectives, and the interactive 3D models can be rotated to enable comprehensive visualization of tooth positions and spatial relationships.

The DICOM files were imported into Dolphin 3D software for image processing and analysis. Image parameters, including brightness, contrast, and opacity, were adjusted to enhance visualization of dental structures. For each subject, the number of erupted permanent teeth, retained primary teeth, and embedded teeth (including unerupted permanent and supernumerary teeth) was recorded (Figure 2) (Digman and Abramowicz 2012; Saunders 2012). Each dental Anomaly was classified by jaw (upper or lower) and types of teeth (anterior teeth, premolars, or molars) (Lu et al. 2024; Tsuji et al. 2020).

In this study, “supernumerary teeth” were defined as additional tooth‐like structures exceeding the normal dental formula, regardless of their eruption status. Conversely, “embedded teeth” referred to permanent teeth and supernumerary teeth that had failed to erupt within the expected time frame and were radiographically retained within the alveolar bone. Notably, supernumerary teeth that fully erupted into the arch were still categorized as supernumerary teeth and did not count among the permanent dentitions (Digman and Abramowicz 2012; Saunders 2012).

### 2.4 Quantitative Variables

Quantitative and categorical variables were handled according to their clinical characteristics and analytical purposes. *RUNX2* variants were categorized into two groups based on the type of structural alteration. Group 1, “Non‐truncating Variants,” comprised missense variants, which result in single amino acid substitutions without causing protein truncation. Group 2, “Truncating/Structural Variants,” included alterations such as microdeletions, frameshift insertions or deletions, and nonsense variants, all of which are predicted to more severely disrupt protein structure. This classification was applied to facilitate comparisons of phenotypic severity between the two groups (Thaweesapphithak et al. 2024). This classification reflects the predicted functional impact of *RUNX2* variants, whereby truncating variants are more likely to result in loss of the C‐terminal proline–serine–threonine (PST) activation domain or undergo nonsense‐mediated mRNA decay, leading to a greater degree of haploinsufficiency, whereas missense variants, particularly those within the Runt domain, may retain partial protein function or exert altered transcriptional activity.

Dental variables were categorized according to tooth type into three groups: anterior teeth (incisors and canines), premolars, and posterior teeth (molars), reflecting their distinct developmental and anatomical characteristics and stage of eruption.

Cephalometric measurements were analyzed as continuous variables and further categorized into three groups (below normal, within normal range, and above normal). This categorization was used to facilitate clinical interpretation and comparison with established craniofacial norms.

### 2.5 Statistical Analysis

All statistical analyses were performed using SPSS version 29.0 (IBM Corp., Armonk, NY, USA). Owing to the small sample size and potential data non‐normality, the Mann–Whitney U test was used to compare cephalometric measurements and dental anomaly counts between variant groups. Spearman correlation analysis assessed relationships among cephalometric parameters.

Dental anomaly distributions across tooth types (anterior teeth, premolars, molars) were compared using the Kruskal–Wallis test, with analyses performed for both combined and stratified (maxilla vs. mandible) jaws. When significant differences were identified, pairwise post hoc comparisons were performed using Dunn's test with Bonferroni correction (Excel‐based). Comparisons between jaws for each tooth type employed the Mann–Whitney U test. Statistical significance was set at *p* < 0.05. No missing data were observed for the primary variables analyzed, as all participants had complete clinical, radiographic, and genetic data sets.

### 2.6 Reliability Assessment

Intra‐examiner reliability was assessed by randomly selecting five cases for repeated analysis of both lateral cephalograms and CBCT scans. All cephalometric landmarks and dental anomaly classifications (embedded, supernumerary, unerupted permanent, and retained primary teeth) were remeasured by the same examiner after a 2‐week interval. Intraclass Correlation Coefficients (ICCs) were calculated in SPSS using a two‐way mixed‐effects model with absolute agreement, and interpreted according to Koo and Li (2016) (Koo and Li 2016), with values < 0.5 indicating poor reliability, 0.5–0.75 moderate, 0.75–0.90 good, and > 0.90 excellent reliability.

### 2.7 Bias and Quality Control

Efforts were made to minimize potential sources of bias. Consecutive patient recruitment was employed to reduce selection bias. All clinical and radiographic assessments were performed using standardized protocols by the same experienced examiner. Genetic analyses were conducted using validated sequencing methods and interpreted according to the ACMG–AMP guidelines. These measures are aimed at reducing measurement and observer bias.

## 3 Results

Participant characteristics such as age, sex, gene mutation, and variant type are summarized in Table 1. No missing data were observed for any variables included in the descriptive or analytical analyses.

### 3.1 Lateral Cephalometric Analysis

The results of the cephalometric analysis, along with their interpretations and *p*‐values comparing the two types of structural alterations (Group 1: missense and Group 2: truncating/structural variants) are presented in Table 2. The cranial base length (CC–Na), the SNA angle, and the nasolabial angle were significantly greater in the missense variant group (Group 1) compared with the truncating/structural variant group (Group 2) (*p* = 0.020 for both). No significant differences were found in the remaining cephalometric parameters.

**TABLE 2:** Comparison of cephalometric measurements between *RUNX2* variant groups.

Measurements	Group 1 Non‐truncating variants						Group 2 truncating/Structural variants						*p*‐value
	Median	Q1 (25th)	Q3 (75th)	IQR	Diff	Interpretation	Median	Q1 (25th)	Q3 (75th)	IQR	Diff	Interpretation	
CC‐Na (mm.)	47.66	47.47	47.97	0.50	−8.34	Short cranial base length	49.58	49.25	51.69	2.44	−6.42	Short cranial base length	0.020*
SNA (°)	92.99	92.56	94.76	2.20	4.39	Prognathic maxilla	86.81	84.54	89.64	5.10	—	Orthognathic maxilla	0.020*
NA‐FH (°)	95.41	94.81	98.3	3.49	1.81	Prognathic maxilla	96.33	93.49	97.64	4.15	2.73	Prognathic maxilla	0.796
SNB (°)	96.61	94.32	97.06	2.74	9.91	Prognathic mandible	90.44	88.45	95.02	6.57	3.74	Prognathic mandible	0.121
SN‐Pog (°)	96.39	95.06	97.14	2.08	11.29	Prognathic mandible	91.22	88.92	95.13	6.21	6.12	Prognathic mandible	0.121
NPog‐FH (°)	98.42	98.02	99.8	1.78	10.22	Prognathic mandible	100.88	99.38	101.51	2.13	12.68	Prognathic mandible	0.302
ANB (°)	−4.49	−4.78	−4.94	−5.34	−5.39	Skeletal Class III	−4.06	−3.95	0.88	1.39	−4.96	Skeletal Class III	1.000
A‐NPog (mm.)	−3.26	−4.20	−2.89	1.31	−5.46	Skeletal Class III	−5.01	−5.51	−2.90	2.61	−7.21	Skeletal Class III	0.796
Wits (mm.)	−7.27	−9.58	−4.38	5.2	−4.24	Skeletal Class III	−9.40	−12.83	−6.32	6.51	−6.37	Skeletal Class III	0.439
NSGn (°)	58.06	57.33	58.33	1.00	−5.04	Skeletal deep bite	59.15	58.62	60.64	2.02	−3.95	Skeletal deep bite	0.197
SN‐GoGn (°)	21.32	20.6	22.74	2.14	−0.78	Skeletal deep bite	22.74	16.17	24.22	8.05	—	Skeletal normal bite	0.796
MP‐PP (°)	19.72	18.84	20.16	1.32	—	Skeletal normal bite	15.69	12.00	19.48	7.48	−0.01	Skeletal deep bite	0.439
LL to E line (mm.)	−2.54	−4.32	−1.58	2.74	−0.54	Retrusive lower lip	−2.02	−3.62	1.41	5.03	−0.02	Retrusive lower lip	0.796
Nasolabial angle (°)	102.66	99.87	113.4	13.53	2.66	Protrusive upper lip	82.58	78.94	87.15	8.21	−7.42	Retrusive upper lip	0.020

Although Group 1 exhibited a significantly longer cranial base than Group 2, values in both groups were still shorter than the Thai normative standard. Regarding maxillary position, the SNA angle indicated a prognathic maxilla in Group 1, while Group 2 showed a normal maxillary position. However, NA‐FH suggested maxillary prognathism in both groups, with no significant difference. For the position of the mandible, measurements of SNB, SN‐Pog, and NPog‐FH confirmed mandibular prognathism in both groups without statistical significance. Consequently, ANB, A to NPog, and Wits appraisal collectively indicated a skeletal Class III relationship in both groups.

No significant differences were observed between the two groups in skeletal vertical relationships, as assessed by NSGn, SN‐GoGn, and MP–PP angles. Both groups demonstrated skeletal deep bite patterns, except that the SN–GoGn angle in the truncating/structural variant group was within the normal range.

For soft tissue analysis, the lower lip position relative to the E‐line was retrusive in both groups, with no significant difference. However, the nasolabial angle was significantly larger in Group 1 (mean 107.96°) than in Group 2 (mean 82.08°), indicating a more protrusive upper lip position in the former (*p* = 0.020), which is consistent with the higher SNA value observed in Group 1.

**FIGURE 3:**
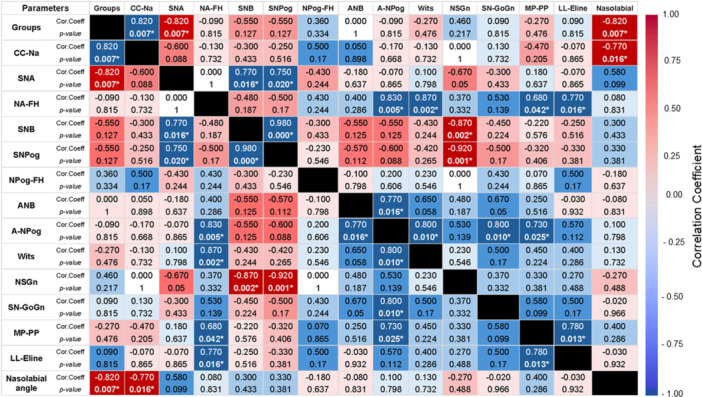
Heatmap illustrating Spearman correlation coefficients among craniofacial and soft tissue measurements. Positive correlations are shown in blue; negative correlations in red. Significant correlations (*p*  <  0.05) were observed between group classification and cranial base length (CC–Na), SNA angle, and nasolabial angle.

Spearman correlation analysis identified several significant associations among craniofacial and soft tissue measurements, as summarized in the heatmap (Figure 3). Group classification was significantly associated with cranial base length (CC–Na), SNA angle, and nasolabial angle (*r* = ±0.822, *p* = 0.007 for all), consistent with the results from the Mann–Whitney U test. In addition, the SNB angle showed a very strong positive correlation with SN–Pog (*r* = 0.983, *p* < 0.01). Wits appraisal was strongly correlated with both Na–FH (*r* = 0.867, *p* = 0.002) and NS–Gn (*r* = –0.867, *p* = 0.002).

Moderate‐to‐strong correlations were also observed between SNB and SN–Pog (*r* = 0.767, *p* = 0.016), nasolabial angle and cranial base length (*r* = –0.767, *p* = 0.016), mandibular plane to palatal plane (MP–PP) and SN–Pog (*r* = 0.683, *p* = 0.042), as well as between the lower lip to E‐line distance and SN–Pog (*r* = 0.767, *p* = 0.016).

### 3.2 Data Collection of Dental Anomalies

The distribution of dental anomalies according to jaw, tooth type, and *RUNX2* variant type is presented in Supporting Information S1: Table 3. No significant difference was observed in any comparison between the two variant groups.

To further explore the potential genotype–phenotype relationship, Table 3 summarizes the specific *RUNX2* mutations identified in each patient alongside their corresponding dental findings, including the number of embedded teeth, supernumerary teeth, unerupted permanent teeth, and retained primary teeth. Considerable variability in dental anomalies was observed among patients harboring the same mutation, while similar phenotypic patterns were also identified across different mutation types. These findings further support the absence of a consistent genotype–phenotype correlation for dental anomalies in this cohort.

**TABLE 3:** Distribution of dental phenotypes among different *RUNX2* variant types.

Patients	Type of variant	Novel	Mutation	Embedded teeth	Supernumerary teeth	Unerupted permanent teeth	Retained primary teeth
**Group 1: Non‐truncating variants**							
Case 2	Missense	Known	c.673 C > T, p.Arg225Trp	21	10	9	12
Case 6	Missense	Novel	c.614 C > T, p.Thr205Ile	31	9	22	34
Case 9	Missense	Novel	c.614 C > T, p.Thr205Ile	8	5	3	3
**Group 2: Truncating/Structural variants**							
Case 1	Microdeletion	Novel	Microdeletion	30	5	25	29
Case 5	Microdeletion	Novel	Microdeletion	33	17	16	23
Case 4	Frameshift InDel	Known	c.739delA, p.Ser247ValfsTer3	32	11	20	30
Case 7	Frameshift InDel	Novel	c.1550_1553del, p.Val517Glyfs*61	26	8	18	28
Case 8	Frameshift InDel	Known	c.90dup, p.Ser31Leufs*130	6	4	5	8
Case 3	Nonsense	Known	c.1081 C > T,p.Gln361Ter	21	10	15	17

**FIGURE 4:**
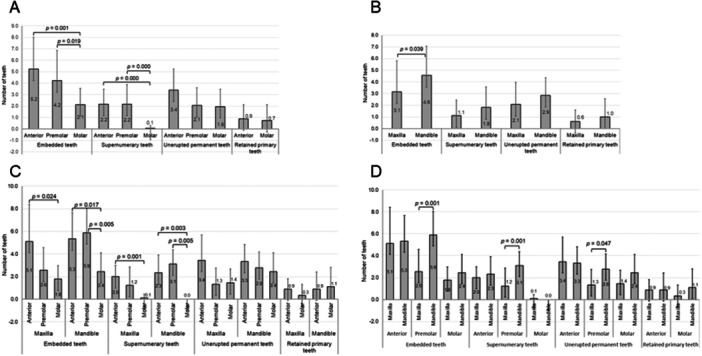
Comparison of dental anomaly counts by tooth type and jaw. (A) Mean number of embedded, supernumerary, unerupted permanent, and retained primary teeth by tooth type (anterior, premolar, molar). (B) Comparison of dental anomalies between the maxilla and the mandible. (C) Distribution of dental anomalies by both tooth type and jaw. (D) Post hoc comparisons of dental anomalies by jaw within each tooth type. Significant differences (*p* <  0.05) are indicated on the graphs.

Figure 4 shows the distribution of dental anomalies by tooth types and jaws. For embedded teeth, the number varied significantly by tooth type, being highest in anterior teeth (mean = 5.2), followed by premolars (mean = 4.2) and molars (mean = 2.1), with significant differences between anterior teeth and molars (*p* = 0.001) and between premolars and molars (*p* = 0.019). For supernumerary teeth, anterior teeth and premolars (mean = 2.2 each) showed significantly higher numbers than molars (mean = 0.1; both *p* < 0.001). The number of unerupted permanent teeth was highest in anterior teeth (mean = 3.4), with significant differences from premolars (mean = 2.1) and molars (mean = 1.9). No significant difference was observed for retained primary teeth among tooth types (Figure 4A).

When comparing by jaw, the number of embedded teeth was significantly greater in the mandible (mean = 4.6) than in the maxilla (mean = 3.1) (*p* = 0.039). No significant difference was found between jaws for supernumerary teeth (maxilla = 1.1, mandible = 1.8), unerupted permanent teeth (maxilla = 2.1, mandible = 2.9), or retained primary teeth (maxilla = 0.6, mandible = 1.0) (Figure 4B).

When analyzed by both jaws and tooth types, the highest number of embedded teeth was observed in the mandibular premolars (mean = 5.9), which was significantly higher than in the maxillary premolars (mean = 2.6; *p* = 0.001). Similarly, supernumerary teeth were significantly more common in the mandibular premolars than in the maxillary premolars (*p* = 0.001). Unerupted permanent teeth were also significantly more prevalent in the mandibular premolars compared to the maxillary premolars (*p* = 0.047). No statistically significant differences were found for retained primary teeth across jaws or tooth types (Figure 4C,D).

### 3.3 Measurement Reliability

Intra‐examiner reliability was assessed using ICC, which demonstrated excellent agreement for all measurements. The ICC values of the cephalometric measurements were as follows: SNA (0.921), NA‐FH (0.925), SNB (0.991), SN–Pog (0.992), NPog‐FH (0.953), ANB (0.991), A to NPog (0.962), Wits appraisal (0.990), N‐S‐Gn (0.993), SN‐GoGn (0.988), MP–PP (0.987), LL to E‐line (0.930), nasolabial angle (0.944), and lower incisor inclination (0.853).

The reliability of dental anomaly assessments demonstrated excellent reliability, with both single and average measure ICCs of 1.000 (95% confidence intervals: 0.999–1.000), indicating near‐perfect agreement and measurement consistency.

## 4 Discussion

This study aimed to investigate the potential correlation between *RUNX2* gene variants and the severity of skeletal Class III malocclusion, as well as the burden of embedded and supernumerary teeth in patients with CCD. Recognizing the phenotypic diversity frequently observed in CCD, particularly with respect to dental and craniofacial anomalies, the study sought to determine whether specific types or locations of *RUNX2* variants could predict the extent of skeletal and dental involvement (Tessa et al. 2003; Thaweesapphithak et al. 2022; Thaweesapphithak et al. 2024; Thaweesapphithak et al. 2023). Unlike prior studies that were largely descriptive or limited to case reports (Chen et al. 2023; Farronato et al. 2009; Park et al. 2013; Rocha et al. 2014), this research employed a systematic approach by integrating genetic analysis with comprehensive radiographic assessments. Radiographic evaluation was conducted using lateral cephalograms and CBCT, allowing for detailed quantification of unerupted permanent teeth, retained primary teeth, embedded teeth, and supernumerary teeth, as well as comprehensive cephalometric analyses. Genetic variants were confirmed through whole exome or Sanger sequencing and categorized by variant type and protein domain affected. These findings suggest that while *RUNX2* variant type may help predict skeletal phenotype severity in CCD, dental anomaly burden appears to be influenced by other factors. Thus, individualized clinical management should account for this variability, and genetic findings alone may not fully inform dental treatment planning.

From a clinical perspective, identifying *RUNX2* variant type may aid in anticipating the severity of skeletal discrepancies, particularly cranial base shortening and maxillary retrognathism, both of which have implications for the timing and modality of orthodontic or orthognathic interventions. In contrast, the unpredictability of dental anomalies reinforces the need for early CBCT imaging and individualized eruption guidance strategies, regardless of genetic findings. These insights emphasize the importance of integrating genetic information with radiographic evaluation for comprehensive, patient‐specific treatment planning in CCD.

Skeletal Class III malocclusion is a frequently observed craniofacial phenotype in patients with CCD (Farronato et al. 2009). This condition primarily results from maxillary hypoplasia leading to midface retrusion, often accompanied by a relatively normal or prognathic mandible, which contributes to a concave facial profile and anterior crossbite (Chen et al. 2023; Farronato et al. 2009; Jensen 1994; Park et al. 2013; Rocha et al. 2014). Midfacial hypoplasia has been closely associated with mutations in the *RUNX2* gene, which encodes a master transcription factor essential for osteoblast differentiation and craniofacial skeletal development (Faria‐Teixeira et al. 2024; Thaweesapphithak et al. 2022; Thaweesapphithak et al. 2024). *RUNX2* mutations, particularly those affecting the Runt homology domain (RHD) and proline–serine–threonine‐rich (PST) regions, impair DNA binding, transactivation, and nuclear localization, ultimately resulting in defective ossification and craniofacial dysmorphology (Thaweesapphithak et al. 2022). Disruptions in key molecular pathways, specifically WNT, BMP, FGF, and Hedgehog (HH) signaling, have been identified as critical mechanisms affecting craniofacial development in CCD. These pathways converge at *RUNX2*, influencing craniofacial skeletal patterning and contributing to the skeletal imbalance observed in affected individuals (Faria‐Teixeira et al. 2024). Thus, CCD exemplifies how gene mutations coupled with upstream pathway dysregulation can result in complex skeletal malformations, highlighting potential molecular targets for future therapeutic intervention.

Opposite to previous studies (Chen et al. 2023; Farronato et al. 2009; Jensen 1994; Park et al. 2013; Rocha et al. 2014), the maxilla in this study appeared to be in a prognathic position in both groups, which may be attributed to the shortened cranial base length. Consistent with Savoldi et al. (2022) (Savoldi et al. 2022), both variant groups in the present study exhibited a reduced anterior cranial base length (CC‐Na), with a mean reduction of 8.34 mm in Group 1 and 6.42 mm in Group 2, potentially influencing the interpretation of anteroposterior jaw positions when applying standard cephalometric norms. Nevertheless, the overall skeletal relationship in this study remained Class III, primarily due to the protruded mandibular position.

Spearman correlation analysis supported the findings from group comparisons, indicating that variant type was significantly associated with both cranial base length (CC–Na) and SNA angle, with the missense variant group showing higher values. This aligns with the Mann–Whitney U test results and suggests that the cranial base may play a role in maxillary positioning in patients with *RUNX2* variants. Strong correlations were also observed among SNB, SN–Pog, and Wits appraisal, highlighting the contribution of mandibular base and chin position to the overall skeletal relationship. In addition, soft tissue parameters, such as the nasolabial angle and lower lip to E‐line distance, were correlated with mandibular skeletal measurements, suggesting an influence of underlying skeletal structure on soft tissue profile. Notably, Wits appraisal showed significant associations with Na–FH and NS–Gn angles, indicating that both the Frankfort horizontal plane and cranial base configuration may impact the cephalometric evaluation of sagittal discrepancy (Savoldi et al. 2022). However, due to the limited sample size, subgroup‐specific correlation analysis was not performed.

Numerous supernumerary teeth, often clustered in the anterior and premolar regions, along with widespread impaction or delayed eruption of the permanent dentition, are characteristic features of CCD (Lu et al. 2024; Shi et al. 2022; Suda et al. 2007; Symkhampha et al. 2021). In this study, embedded and supernumerary teeth were categorized by tooth type rather than by region, as multiple embedded teeth are often closely packed, making regional classification difficult. Anterior teeth and premolars were differentiated based on the number of cusps. In this study, supernumerary teeth and unerupted permanent teeth (included in the embedded teeth category) were predominantly found in the anterior teeth and premolars. Notably, no supernumerary molars were observed in any group; all embedded molars were unerupted permanent teeth.

Previous studies by Suda et al. in 2007 (Suda et al. 2007) and 2010 (Suda et al. 2010) examined the relationship between *RUNX2* variants and supernumerary tooth formation in CCD. The 2007 study analyzed three siblings with the same *RUNX2* variant (P210S) and found intrafamilial variability in the number, location, and symmetry of supernumerary teeth. In the 2010 study, *RUNX2* mutations were identified in 9 individuals with CCD from 5 Japanese families using Sanger sequencing. Although some patients shared identical mutations, such as R225Q or P224S, the number and position of supernumerary teeth varied substantially, even among individuals within the same family. Notably, monozygotic twins carrying the same mutation also exhibited differences in the number and location of supernumerary teeth. A study by Bufalino et al. (2012) involving 11 CCD patients reported that individuals with missense mutations in the *RUNX2* runt domain (e.g., R190Q, R225Q) tended to exhibit more severe dental anomalies, including a higher number of supernumerary and impacted teeth, compared to those without *RUNX2* mutations or with mutations outside the Runt domain. However, considerable phenotypic variability was observed even among individuals with the same mutation, including within families (Bufalino et al. 2012). Consistent with these findings, the present study did not reveal a clear correlation between mutation type and the burden or distribution of supernumerary teeth. This is further supported by the individual‐level analysis presented in Table 3, which directly compares specific *RUNX2* mutations with corresponding dental anomalies in each patient. Notably, substantial variability in dental findings was observed among individuals harboring the same mutation, while similar phenotypic patterns were also identified across different mutation types. These observations indicate the absence of a consistent genotype–phenotype relationship for dental anomalies and suggest that factors beyond *RUNX2* mutations, such as epigenetic influences, environmental factors, or local eruption conditions, may contribute to the observed heterogeneity in CCD.

The relatively lower ICC values observed for SNA, NA‐FH, and ANB (0.921, 0.925, and 0.920, respectively) can be attributed to the anatomical characteristics of point A, which is a common reference point in all 3 measurements. In this cohort, superimposition of embedded teeth in the anterior maxilla frequently obscured the clear identification of point A on the lateral cephalometric radiographs. This overlapping of dental structures likely introduced variability in landmark localization, thereby reducing the reproducibility of these cephalometric parameters. Nevertheless, all 3 ICC values remained above 0.90, indicating excellent reliability.

This study has several limitations that should be acknowledged. First, a key limitation is the small sample size, which reflects the rarity of CCD, with an estimated prevalence of approximately one in 1,000,000 individuals worldwide (Machol et al. 2006; McNamara et al. 1999), Of the 15 affected individuals, only 9 met the inclusion criteria for radiographic analysis. Three younger patients were excluded to ensure adequate mandibular skeletal maturity (Burstone 1963; Manlove et al. 2020), and 2 individuals with a history of tooth extraction or surgical removal of embedded or supernumerary teeth were excluded to preserve the accuracy of dental anomaly assessments. Thus, a total of 6 individuals were excluded from the study, which has been reflected in the flow diagram (Figure 1). Although these strict inclusion criteria enhanced the reliability of craniofacial evaluations, they further reduced the sample size and may also limit the generalizability of the findings. Clinic‐based recruitment may have introduced selection bias, potentially overrepresenting more severe CCD phenotypes compared with community‐based populations. The small sample size may also have reduced statistical power and increased estimation imprecision, which could lead to either underestimation or overestimation of true effect sizes. Therefore, the findings of this study should be interpreted as preliminary observations rather than definitive evidence of genotype–phenotype correlation. Although high intra‐examiner reliability was demonstrated, minor measurement bias cannot be entirely excluded; however, given the excellent ICC values, the magnitude of this bias is likely to be low.

Second, subgroup analyses based on specific *RUNX2*‐variant locations or functional domains were not feasible due to the limited number of individuals included in the radiographic analysis. As CCD is a rare disorder, the small sample size limited our ability to perform mutation‐specific or domain‐specific genotype–phenotype analyses, which may have obscured more subtle correlations. Third, due to this limitation, *RUNX2* variants were grouped into 2 broader categories, non‐truncating (missense) and truncating/structural variants, to enable meaningful statistical comparison. Although this classification reflects the predicted functional impact of the variants, it may oversimplify the biological heterogeneity of *RUNX2* mutations. Variants occurring in different functional domains, such as the Runt domain or the PST region, may lead to distinct phenotypic effects even within the same variant category. Finally, this study consisted exclusively of Thai individuals and was conducted in a single tertiary referral center, which may limit the external validity of the findings. Therefore, caution should be exercised when generalizing these results to broader populations or different healthcare settings. Future multicenter studies with larger cohorts will be necessary to determine whether domain‐specific or mutation‐specific genotype–phenotype correlations exist in CCD.

In summary, this study demonstrates a potential genotype–phenotype correlation in skeletal characteristics, but not in dental anomalies, among patients with CCD harboring *RUNX2* variants. These findings highlight the multifactorial nature of dental development and suggest that genetic analysis alone may be insufficient to predict the burden of dental anomalies. To the best of our knowledge, this is the first study to systematically investigate genotype–phenotype correlations in CCD by integrating molecular classification with comprehensive radiographic analysis. This combined approach offers novel insights into the craniofacial variability associated with *RUNX2* mutations and underscores the potential for genotype‐informed orthodontic and surgical treatment planning. Given the rarity of CCD and the complexity of its phenotypic expression, further multicenter studies involving larger, ethnically diverse cohorts and domain‐specific variant analyses are warranted to validate these correlations, identify underlying genetic or environmental modifiers, and improve prediction accuracy to guide early, individualized clinical interventions.

## 5 Conclusion

This study demonstrated that the *RUNX2* variant type is significantly associated with cranial base length and maxillary position, thereby influencing the severity of skeletal Class III malocclusion in patients with CCD. Clinically, these findings underscore the importance of integrating genetic analysis into orthodontic and surgical treatment planning, particularly for anticipating skeletal discrepancies. In contrast, the burden and distribution of embedded and supernumerary teeth were not correlated with *RUNX2* variant type, as demonstrated by both group‐level analyses and individual‐level comparisons. These findings indicate the absence of a consistent genotype–phenotype relationship for dental anomalies and suggest the influence of additional genetic, epigenetic, or environmental factors. Therefore, individualized treatment approaches remain essential.

## Author Contributions

Pintu‐on Chantarawaratit contributed to the conception and design of the study, data acquisition, formal analysis, and interpretation of data, and drafted and critically revised the manuscript. Sermporn Thaweesapphithak contributed to sample collection, data acquisition, formal analysis, and interpretation of data. Pisha Pittayapat contributed to sample collection, data acquisition, and interpretation of data. Thantrira Porntaveetus contributed to the conception and design of the study, coordination of the research, data analysis, interpretation of data, funding acquisition, and critical revision of the manuscript. All authors have materially participated in the research and/or article preparation, provided final approval of the submitted version, and agreed to be accountable for all aspects of the work, ensuring its integrity and accuracy.

## Ethics Statement

This study was approved by the Human Research Ethics Committee, Faculty of Dentistry, Chulalongkorn University (HREC‐DCU 2021‐030, 2025‐141) and conducted in accordance with the Declaration of Helsinki.

## Consent

Written informed consent was obtained from all participants or their legal guardians prior to enrollment in the study. No previously published figures, tables, or text requiring copyright permission were reproduced in this manuscript.

## Conflicts of Interest

The authors declare no conflicts of interest.

## Supporting information

Supporting File 1

Supporting File 2

## Data Availability

The data sets generated and/or analyzed during the current study are available from the corresponding author on reasonable request. Sequencing data are not publicly available due to privacy restrictions on human genetic information.
