# Supplementary material for: Correlation of RUNX2 Variants With Craniofacial–Dental Phenotypes in Cleidocranial Dysplasia
Source: Clin Exp Dent Res. 2026 Apr 17;12(2):e70351. doi: 10.1002/cre2.70351 (PMC13088869; doi:10.1002/cre2.70351)
Supplement: Supplementary file 1 — Supporting File 1 [file CRE2-12-e70351-s002.docx]

**Supplementary Table 1** Definitions of the cephalometric landmarks used in this study (Duangsuwan, Raocharernporn, Thiradilok, & Manopatanakul, 2023; Kapila & Nervina, 2015)

| **Name** | **Anatomical definition** |
| --- | --- |
| Sella turcica (S) | Anteroposterior midpoint of the pituitary fossa of the sphenoid bone |
| Nasion (N) | Most anterior point of the frontonasal suture |
| Basion (Ba) | The lowest point on the anterior margin of the foramen magnum in the median plane |
| Pterygoid point (Pt) | Posterior-most and superior-most point in the upper contour of the pterygomaxillary fissure. |
| Center of Cranium (CC) | A cephalometric landmark defined as the point of intersection between the Ba–Na (Basion–Nasion) line and the Pt–Gn (Pterygoid point–Gnathion) line. It represents the geometric center of the cranium in the sagittal plane, as used in Ricketts analysis. |
| Point A (A) | Most posterior point of the maxillary curvature, between the anterior nasal spine and the supradental point |
| Point B (B) | Most posterior point of the anterior surface of the mandibular symphysis |
| Anterior nasal spine (ANS) | Most anterior point of the maxillary process in the nasal floor region |
| Posterior nasal spine (PNS) | Most posterior and midpoint of the palatine bone contour |
| Gnathion (Gn) | Most anteroinferior point of the mandibular symphysis |
| Gonion (Go) | The posterior-inferior point at the mandibular angle, where the posterior ramus meets the lower border of the mandible, identified by bisecting the angle between them. |
| Pogonion (Pog) | Most anterior point on the contour of the bony chin |
| Frankfort horizontal plane (FH) | A plane extending from the porion (Po) and the orbital (Or) |
| Palatal plane (PP) | A plane extending from the anterior nasal spine (ANS) to the posterior nasal spine (PNS). |
| Mandibular plane | A plane extending from the gonion (Go) to the gnathion (Gn). |

**Supplementary Table 2** Lateral Cephalometric Measurements and Normative Values in a Thai Population (Duangsuwan, Raocharernporn, Thiradilok, & Manopatanakul, 2023; Sorathesn, 1988)

| **Area** | **Measurements** | **Description** | **Thai Norm** | | **Diagnostic Implication** |
| --- | --- | --- | --- | --- | --- |
|  |  |  | **Mean** | **SD** |  |
| Anterior cranial base length | CC-Na (mm.) | The linear distance between CC (Center of Cranium) and Na (Nasion). | 58.7 | 2.7 | Decreased distance suggests a short anterior cranial base.  Increased distance suggests an elongated anterior cranial base. |
| Maxillary position relative to cranial base  (Antero-posterior) | SNA (°) | Angle between the sella–nasion line (SN) and the nasion–A point line | 85.0 | 3.6 | Decreased angle suggests maxillary retrusion; increased angle suggests maxillary protrusion. |
|  | NA-FH (°) | Angle between the nasion–A point line and the Frankfort horizontal plane (FH; Or-Po) | 90.3 | 3.3 | Decreased angle suggests maxillary retroclination; increased angle suggests maxillary proclination. |
| Mandibular position relative to cranial base (Antero-posterior) | SNB (°) | Angle between the sella–nasion line (SN) and the nasion–B point line | 82.3 | 4.4 | Decreased angle suggests mandibular retrusion; increased angle suggests mandibular prognathism. |
|  | SN-Pog (°) | Angle between the sella–nasion line (SN) and the sella–pogonion line | 82 | 3.09 | Decreased angle suggests mandibular retrusion; increased angle suggests mandibular protrusion. |
|  | NPog-FH (°) | Angle between the nasion–pogonion line (N–Pog) and the Frankfort horizontal plane (FH; Or-Po) | 85 | 3.2 | Decreased angle suggests a retrusive chin or flat profile; increased angle suggests mandibular prominence. |
| Maxillo-mandibular  relationship  (Antero-posterior) | ANB (°) | Angle between A point, nasion, and B point | 2.8 | 1.9 | Decreased angle suggests skeletal Class III; increased angle suggests skeletal Class II. |
|  | A-NPog (mm.) | Linear distance between point A and nasion–pogonion line (NPog) | 4.5 | 2.3 | Decreased distance suggests skeletal Class II; increased distance suggests skeletal Class III. |
|  | Wits (mm.) | Distance between perpendicular projections of points A and B onto the functional occlusal plane | -3 | 2 | Negative value suggests skeletal Class III; positive value suggests skeletal Class II. |
| Vertical skeletal pattern | NSGn (°) | Angle between nasion–sella line (NS) and sella–gnathion line (SGn) | 67.4 | 4.3 | Decreased angle suggests horizontal growth pattern; increased angle suggests vertical growth pattern. |
|  | SN-GoGn (°) | Angle between the sella–nasion line (SN) and the mandibular plane (Go–Gn) | 29.4 | 7.3 | Decreased angle suggests skeletal deep bite tendency; increased angle suggests skeletal open bite tendency. |
|  | MP-PP (°) | Angle between the mandibular plane (MP) and the palatal plane (PP) | 21 | 5.25 | Decreased angle suggests skeletal deep bite tendency; increased angle suggests skeletal open bite tendency. |
| Soft tissue | LL to E line (mm.) | Linear distance from the lower lip to Ricketts’ esthetic line (E-line) | 3.5 | 2 | Decreased distance suggests a retrusive lower lip position; increased distance suggests lower lip protrusion beyond the esthetic line. |
|  | Nasolabial angle (°) | Angle formed between the columella and the upper lip | 90 | 9 | Decreased angle suggests upper lip protrusion; increased angle suggests maxillary retrusion. |

**Supplementary Table 3** Distribution of Embedded, Supernumerary, Unerupted Permanent and Retained Primary Teeth According to Jaws, Type of Teeth, and Types of *RUNX2* Variants

| Dental Anomalies | Jaws | Types of Teeth | Types of *RUNX2* variants | | | | | | | | | | | Total  (N = 9) | | *p*-value  (Group 1 VS Group 2) | *p*-value  (Group 1 VS Group 2) |
| --- | --- | --- | --- | --- | --- | --- | --- | --- | --- | --- | --- | --- | --- | --- | --- | --- | --- |
|  |  |  | Group 1  Non-truncating variants | | Group 2  Truncating/Structural variants | | | | | | | | |  |  |  |  |
|  |  |  | Missense  (N = 3) | | Microdeletion  (N = 2) | | Frameshift InDel  (N = 3) | | Nonsense  (N = 1) | | | Total  (N = 6) | |  |  |  |  |
|  |  |  | Mean | SD | Mean | SD | Mean | SD | Tooth count | SD | Mean | | SD | Mean | SD |  |  |
| Embedded teeth | Maxilla | Anterior | 4.0 | 5.7 | 6.0 | 2.8 | 5.0 | 4.2 | 5 | - | 6.0 | | 2.8 | 5.1 | 3.3 | .751 | .374 |
|  |  | Premolar | 2.5 | 2.1 | 5.0 | 1.4 | 0.5 | 0.7 | 1 | - | 2.7 | | 2.3 | 2.6 | 2.0 |  |  |
|  |  | Molar | 2.5 | 2.1 | 1.5 | 0.7 | 1.0 | 1.4 | 2 | - | 1.3 | | 0.8 | 1.8 | 1.2 |  |  |
|  | Mandible | Anterior | 4.0 | 1.4 | 7.5 | 0.7 | 3.5 | 3.5 | 4 | - | 5.3 | | 2.5 | 5.3 | 2.3 | .324 |  |
|  |  | Premolar | 4.5 | 2.1 | 7.5 | 2.1 | 5.0 | 2.8 | 7 | - | 6.7 | | 2.1 | 5.9 | 2.1 |  |  |
|  |  | Posterior | 2.0 | 2.8 | 4.0 | 0.0 | 1.0 | 1.4 | 2 | - | 2.7 | | 1.6 | 2.4 | 1.7 |  |  |
| Supernumerary teeth | Maxilla | Anterior | 1.5 | 2.1 | 2.0 | 0.0 | 2.5 | 0.7 | 2 | - | 2.3 | | 0.5 | 2.0 | 1.0 | .891 | .992 |
|  |  | Premolar | 1.0 | 0.0 | 2.5 | 3.5 | 0.0 | 0.0 | 0 | - | 1.2 | | 2.0 | 1.2 | 1.6 |  |  |
|  |  | Molar | 0.0 | 0.0 | 0.0 | 0.0 | 0.0 | 0.0 | 0 | - | 0.0 | | 0.0 | 0.1 | 0.3 |  |  |
|  | Mandible | Anterior | 2.0 | 1.4 | 3.0 | 2.8 | 1.0 | 1.4 | 3 | - | 2.2 | | 1.7 | 2.3 | 1.6 | .894 |  |
|  |  | Premolar | 2.5 | 0.7 | 3.5 | 2.1 | 2.5 | 0.7 | 5 | - | 3.5 | | 1.4 | 3.1 | 1.3 |  |  |
|  |  | Molar | 0.0 | 0.0 | 0.0 | 0.0 | 0.0 | 0.0 | 0 | - | 0.0 | | 0.0 | 0.0 | 0.0 |  |  |
| Unerupted permanent teeth | Maxilla | Anterior | 2.5 | 3.5 | 4.0 | 2.8 | 3.5 | 2.1 | 4 | - | 4.2 | | 1.8 | 3.4 | 2.2 | .181 | .096 |
|  |  | Premolar | 1.5 | 2.1 | 2.5 | 2.1 | 0.5 | 0.7 | 1 | - | 1.5 | | 1.4 | 1.3 | 1.4 |  |  |
|  |  | Molar | 2.5 | 2.1 | 1.5 | 0.7 | 1.0 | 1.4 | 2 | - | 1.3 | | 0.8 | 1.4 | 1.2 |  |  |
|  | Mandible | Anterior | 2.0 | 0.0 | 4.5 | 2.1 | 2.5 | 2.1 | 4 | - | 3.7 | | 1.6 | 3.3 | 1.5 | .147 |  |
|  |  | Premolar | 2.0 | 2.8 | 4.0 | 0.0 | 3.0 | 1.4 | 2 | - | 3.2 | | 1.0 | 2.8 | 1.4 |  |  |
|  |  | Molar | 2.0 | 2.8 | 4.0 | 0.0 | 1.0 | 1.4 | 2 | - | 2.7 | | 1.6 | 2.4 | 1.7 |  |  |
| Retained  primary teeth | Maxilla | Anterior | 0.5 | 0.7 | 0.0 | 0.0 | 2.0 | 0.0 | 0 | - | 1.0 | | 1.1 | 0.9 | 0.9 | .401 | .936 |
|  |  | Posterior | 1.5 | 2.1 | 0.0 | 0.0 | 0.0 | 0.0 | 0 | - | 0.0 | | 0.0 | 0.3 | 1.0 |  |  |
|  | Mandible | Anterior | 0.0 | 0.0 | 0.0 | 0.0 | 2.0 | 1.4 | 0 | - | 1.3 | | 1.8 | 0.9 | 1.5 | .433 |  |
|  |  | Molar | 2.0 | 2.8 | 1.5 | 2.1 | 1.5 | 2.1 | 0 | - | 1.0 | | 1.5 | 1.1 | 1.7 |  |  |
